# Osteonecrosis of the Jaws (ONJ) after Bisphosphonate Treatment in Patients with Multiple Myeloma: Decreasing ONJ Incidence after Adoption of Preventive Measures

**DOI:** 10.3390/dj4040045

**Published:** 2016-12-01

**Authors:** Gioacchino Catania, Federico Monaco, Giulia Limberti, Manuela Alessio, Iolanda De Martino, Cecilia Barile, Antonella Fasciolo, Anna Baraldi, Marco Ladetto, Vittorio Fusco

**Affiliations:** 1 Haematology Unit, Alessandria Hospital, 15121 Alessandria AL, Italy; 2 Oncology Unit, Alessandria Hospital, 15121 Alessandria AL, Italy; 3 Centro Documentazione Osteonecrosi, Alessandria Hospital, 15121 Alessandria AL, Italy; 4 Maxillofacial Unit, Alessandria Hospital, 15121 Alessandria AL, Italy

**Keywords:** Bisphosphonate, pamidronate, zoledronic acid, Multiple Myeloma, Osteonecrosis of the Jaws, BRONJ, MRONJ, prevention

## Abstract

Bisphosphonates (BPs) are administered to Multiple Myeloma (MM) patients with bone lytic lesion. Osteonecrosis of the Jaw (ONJ) is a complication reported since 2003 in patients treated with intravenous (IV) BPs such as zoledronic acid and pamidronate, with 6%–26.3% frequency in early literature series, before some preventive measures were recommended. We evaluated the occurrence of ONJ with and without dental preventive measures in MM patients treated with BPs in our centre between 1996 and 2015. Since 2005, MM patients (already under treatment or before treatment) underwent a baseline mouth assessment (dental visit, Rx orthopantomography, and eventual tooth avulsion or dental care if necessary) and were followed by a multidisciplinary team. We reviewed the charts of 119 MM patients receiving IV BPs, classified into 3 groups: (*a*) “historic group” (21 patients who had started BP treatment in years before the awareness of ONJ); (*b*) “screening group” (20 patients starting BPs without baseline evaluation); and (*c*) “prevention group” (78 patients starting therapy only after baseline preventive assessment and eventual dental care measures). ONJ was observed in 3/21 patients (14.2%) from group *a*, in 2/20 patients (10%) from group *b*, and in no patients from group *c* (0%). Notably, the median number of IV BP administrations decreased after 2005. Our data confirmed a meaningful reduction of ONJ risk in MM patients treated with BPs if preventive measures are applied. Both implementation of prevention measures and reduction of cumulative doses of IV BPs could have contributed to a decreased incidence of ONJ.

## 1. Introduction

Multiple myeloma (MM) is a haematological cancer characterized by plasma cell proliferation associated with an increased level of monoclonal proteins in blood and/or urine. MM accounts for 1% of all cancer and 10% of all haematological malignancies. The incidence in Europe is 4.5–6/100,000/year with a median age at diagnosis between 65 and 70 years [1]. Until recently, the presence of end-organ damage that causes signs and symptoms of disease (hypercalcaemia, renal failure, anemia, or bone lesions) defined the MM [2]. 

In MM patients, bone lesions are the primary cause of bone pain, which is one of the most common symptoms. About 80% of patients diagnosed with MM have some degree of bone loss. Bone destruction by osteolytic lesions is caused by two separate events: inhibition of normal bone-forming by myeloma cells, and increased activity of osteoclast cells. 

Therapy with bisphosphonates (BPs) is recommended for MM patients who have bone lesions appearing as osteolytic on imaging studies and/or spinal cord compression [3]. Intravenous (IV) BPs—especially pamidronate and zoledronic acid—are strong inhibitors of osteoclast-mediated bone resorption, and it is well accepted that tumour cells in bone can stimulate osteoclast formation and activity, leading to the release of growth factors or cytokines which will further stimulate cancer cell growth and the secretion of osteolytic factors. The prolonged use of IV BPs can result in the development of osteonecrosis of the jaws (ONJ), classically defined as “exposed bone in the maxillofacial region that has persisted for more than 8 weeks in patients with a history of BP treatment and no history of radiation in the jaw region”. Clinical signs may include healing disturbances, inflammations, infections, fistulas, dysesthesiae, and pain [4]. The pathogenesis of bisphosphonate-related osteonecrosis of the jaws (BRONJ) remains unclear, though the suppression of osteoclast mediated bone remodelling with consequent bone sclerosis and ischemia has been suggested as the likely main causal mechanism. In the early retrospective studies, the incidence of BRONJ in MM patients was estimated to be between 1% and 24% of the treated patients [5,6,7,8,9,10,11,12]. In cancer populations, the most important risk factors are: the type of BP (zoledronic acid in comparison with pamidronate), the length of BP treatment, the cumulative BP dose, local infections, and dental procedures [13,14]. The implementation of appropriate dental preventive measures greatly reduced the number of BRONJ cases in one study on MM patients (26.3% vs. 6.7%, p 0.002) [15]. The aim of our study was to evaluate the incidence of BRONJ in one mono-centric experience in MM patients treated with IV BPs, with and without preventive measures. 

## 2. Results

### 2.1. Patients and Methods

We have evaluated MM patients who received BP therapy for bone lesions at our hospital from April 1996 to December 2015. All patients received therapy with intravenous pamidronate (90 mg every 3–4 weeks) and/or zoledronic acid (4 mg every 3–4 weeks), with or without other bisphosphonates (oral or intravenous clodronate; oral alendronate; oral risedronate) in succession along their disease history. 

On December 2005, after observation of first cases of ONJ at our centre, the Alessandria Hospital Oncology-Haematology Department created a multidisciplinary team to manage the emergence and future risk of ONJ, including dentists, maxillofacial surgeons, haematologists, oncologists, nurses, radiologists, nuclear medicine specialists, and infectious disease specialists.

All of the patients already on BP treatment were evaluated by dentists looking for clinical signs of ONJ (bone exposure, pain, fistulae, etc.) and underwent Rx orthopantomography. For patients who were eligible to start BP treatment, the team introduced preventive measures recommended by literature and aimed at reducing the risk of ONJ. These were pre-therapy oral assessment (including dental visit and orthopantomogram), eventual tooth avulsion or dental care to treat existing inflammatory diseases and to prevent invasive oral procedures during the BP treatment, and planned dental visits during BP therapy and after discontinuation. According to clinical conditions, BP treatment was delayed for 1–2 months in the presence of invasive procedures or severe local infections needing therapy. 

According to preventive measures, patients were divided into three groups: (*a*) “*historic group*”, patients starting BP treatment in years 1996–2004, before ONJ literature appeared (with no baseline assessment, not recommended at that time); (*b*) “*screening group*”, patients starting BPs without preventive measures during 2005, or in the following years (in case of immediate need of BP therapy), all followed by the multidisciplinary team; and (*c*) “*prevention group*”, patients who started therapy only after preventive visit and eventual preventive measures (Table 1). 

All patients signed informed consent before continuing (“historic” and “screening” groups) or starting (“*prevention*” group) BP therapy, and they received a letter reporting about ONJ risk to be delivered to practitioners and dentists.

Collected patient data included:

(a) demographics: age, sex;
(b) myeloma history (date of diagnosis; systemic therapies, including antiangiogenic drugs);
(c) BP therapy: start date; treatment duration; type of first-line BP, dosage, and number of administrations; eventual second- and third-line therapy in case of switch to other BPs;
(d) follow-up (from the first BP administration to the latest visit, or death);
(e) possible ONJ risk factors: dental comorbidities or possible precipitating events, such as teeth extraction, periodontal surgery, dental implants, or traumatic use of dentures;
(f) clinical findings at the ONJ diagnosis; ONJ treatment and evolution.

Data were elaborated by the SPSS v.18 system.

### 2.2. Observed Data

Charts of 119 MM patients receiving IV BPs between 1996 and 2015 were reviewed. Patients were classified into three groups, defined as follows: 21 patients in the “*historic group*”, 20 patients in the “*screening group*”, and 78 patients in the “*prevention group*”.

The main data of the patients of the three groups are illustrated in Table 1. Sex and age distribution were similar in the three groups, but differences were present in the range of number of IV administrations (higher in the “*historic*” group) and in the type of BP drug administered as first- or second-line therapy (prevalence of pamidronate in first line and zoledronic acid as second line in the “*historic*” group; the reverse in the “*screening*” group; slight predominance of pamidronate as first line in the third group). 

The median follow-up time was 63.4 months (range 20.3–240.0) in group *a*, 30.2 months (4.4–126.3) in group *b*, and 47.4 months (range 2.8–124.8) in group *c*.

ONJ (defined as the presence of bone exposure, or signs and symptoms with severe computed tomography jawbone alterations) was observed in 3/21 patients (14.2%) in group *a*, in 2/20 patients (10%) in group *b*, and in none patient from group *c* (0%). The main characteristics of every single ONJ patient are illustrated in Table 2. The global crude frequency of ONJ in groups *a* and *b* was 5/41 (12.2%). Ten patients in the prevention group were submitted to preventive teeth extraction without developing ONJ.

The restricted number of cases precluded evaluation of the actuarial risk of ONJ (after two, three and four years from the start of BP treatment, and so on) in the “*historic*” and in the “*screening*” groups. 

## 3. Discussion

### 3.1. ONJ in MM Patients: Literature Data

Osteonecrosis of the jaw (ONJ) is a side effect of BPs firstly recognized in 2003, and whose awareness largely diffused only after 2005 [4]. 

Intravenous BPs (mainly clodronate and pamidronate) had become standard treatment of metastatic bone lesions in cancer and MM patients in the 1990s. Since 2001, zoledronic acid was largely introduced after a single trial comparing pamidronate with zoledronic acid in breast cancer and myeloma patients [16,17]; consequently, many patients receiving pamidronate were shifted to zoledronic acid. In 2002–2003 the Ameican Society Clinical Oncology (ASCO) guidelines for MM and breast cancer patients recommended prolonged BP therapy (indefinitely “*until there is evidence of a substantial decline in a patient’s performance status*”) [18,19]. 

Patients suffering from MM were largely represented in the first cases of reported ONJ after 2003: they were 18 out of 36 ONJ cases observed by Marx [20], and 28 patients out of 63 ONJ cases in the paper by Ruggiero et al. [21]. At the 2004 American Society of Haematology (ASH) meeting, Durie et al. reported the preliminary results of a web-based survey among MM patients (due to collaboration with International Myeloma Foundation, a patient advocacy group) [22]. However, awareness increased largely only after a publication in the New England Journal of Medicine in July 2005 [5]. 

Since then, measures aimed at reducing the risk of ONJ were empirically advised [23,24,25,26], and were diffused among both oncology and haematology professionals and oral care specialists.

The frequency of ONJ ranged between 3.5% and 24.4% in the main retrospective case series of ONJ in MM patients published in years 2005–2006 (illustrated in Table 3) [5,6,7,8,9,10,11,12]. In a Cochrane review of three randomized trials and nine observational studies, ONJ frequency in MM patients ranged between 0 and 51% [27].

Globally, risk factors for ONJ appeared to be both local (dental infections, tooth extractions, oral surgery, ill-fitting dentures) and systemic (number of BP administrations; BP treatment duration; type of BP, with higher risk for zoledronic acid vs. pamidronate vs. other BPs). The possible role of antiangiogenic agents (i.e., thalidomide) in MM patients was not confirmed, whereas it seems more important in cancer patients [14]. 

Since 2006, the scenario of the BP treatment of MM patients radically changed, as a consequence of the emerging ONJ phenomenon and increasing data about the renal toxicity of zoledronic acid: several MM guidelines modified recommendations towards shorter and tailored therapies [3,28,29,30,31]. Suggestions—which were not uniform among the guidelines—included: one or two years of monthly BPs and then discontinuing in patients with responsive or stable disease; eventual infusions every three months after one or two years of monthly therapy; favouring pamidronate (or even clodronate) versus zoledronic acid; dental preventive measures (whenever possible before starting BP therapy); 

On 2009, Dimopoulos et al. [15] published the first report demonstrating the apparent efficacy of preventive measures on ONJ frequency, reduced from 26.3% to 6.7% in a population of MM patients receiving zoledronic acid, coupling another report in metastatic cancer patients [32]. 

In a Nordic randomized trial on 382 MM patients evaluated for ONJ, pamidronate at two different doses (30 mg vs. 90 mg) determined, respectively, 2 and 8 ONJ cases, confirming the role of cumulative BP dose as an ONJ risk factor [33]. 

In 2010, Morgan et al. [34] reported results of a complex randomized trial of comparison between oral 1600 mg daily clodronate and IV 4 mg zoledronic acid every 3–4 weeks in 1960 MM patients recruited between 2003 and 2007. ONJ was reported in 35 patients after zoledronic acid (4%) and in 3 patients after clodronate (<1%), after a median drug exposure of 350 days and a median follow-up of 3.7 years. Unfortunately, no measure of effect of preventive measures are deducible from that report: the Authors stated that they “followed the recommendations of Weitzman and colleagues” (written in 2006, following a Novartis 2004 document, and published in 2007) [27] to reduce the risk of ONJ and to identify and manage such cases. As they enrolled all patients between May 2003 and November 2007, there should be a landmark time point after which the trialists became aware of the clinical definition of ONJ and of the possibility of pre-therapy dental preventive measure, so that a differentiated ONJ frequency evaluation could have hypothetically been drawn, but these data were not reported in the original paper. Further, an estimate of the cumulative ONJ incidence—accounting for competing events (such as death)—would have been more informative, but was lacking. In a more recent safety update report of that trial [35], after a median follow-up of 5.9 years, the ONJ occurrence data were similar (36 ONJ cases after zoledronic acid and 5 after clodronate), whereas interesting data were added. The authors specified that oral health recommendations had been provided to investigators from June 2006. Most ONJ events occurred between 8 and 30 months (median time to ONJ was 23.7 months, with a large range: 8.5–69.5 months). Cumulative incidence figures were presented, suggesting 3%–7% of ONJ risk after 24–36 months in the zoledronic acid arms, and much lower risk for patients receiving clodronate. The authors specified that switching agent at the discretion of the local investigator was more likely later on, and data were not collected. Surprisingly, the incidence of ONJ was numerically lower among patients receiving treatment regimens containing thalidomide compared with the non-thalidomide regimens (1.4% vs. 2.76%, respectively; *p* = 0.041). Unfortunately, a differentiated ONJ frequency estimation between patients recruited before and after June 2006—that is, with or without pre-therapy preventive oral care measures—was again lacking [35]. 

### 3.2. ONJ in MM Patients: The Present Study

Our case series reflects the worldwide changing scenario of BP therapy in MM patients between 2003 and today. At our centre, up to 2005, MM patients were submitted to long-term pamidronate therapy, and many were shifted to zoledronic acid after its introduction to the armamentarium: in this “*historic group*”, 3 ONJ cases out of 21 patients (14.2%) were registered after our hospital specialists were informed about the risk of this “new” disease in MM patients. “*Historic group*” patients received a high number of infusions (median 20, with a range up to 102 administrations), and ONJ patients developed oral disease respectively after 68 (all pamidronate), 102 (79 pamidronate plus 23 zoledronic acid), 75 (all pamidronate) BP infusions. 

After our hospital oncologists and haematologists prompted the establishment of a multidisciplinary team at the end of 2005, the oral health of MM patients was more strictly followed. Twenty patients already under BP treatment for a few months or starting without pre-start preventive measures (due to urgent BP treatment according to the treating physician evaluation) were included in a “*screening group*”, which predominantly received zoledronic acid as first line BP and had a lower median number of infusions (13.5, with range 1–52); in the following years, 2 patients out 20 (10%) developed ONJ, after 3 and 18 zoledronic acid administrations, respectively. 

In more recent years, 78 MM patients were submitted to dental evaluation before the start of BP therapy and had eventual dental care if necessary, in order to minimize the risk of dental disease and required oral surgery during BP treatment, according to on-going recommendations [3,23,24,25,26,28,29,30,31]. MM patients in this “*preventive*” group to this point have received a shorter BP treatment (median 13 infusions, with range 1–37), represented in the first line by zoledronic acid in 36 patients (10 then switching to pamidronate) and pamidronate in 41 patients (2 switching to zoledronic acid). None of these patients have yet developed ONJ.

Our study seems to confirm the value of oral health preventive measures to minimize the risk of ONJ in MM patients submitted to BP therapy. The three ONJ cases observed in our “*historic*” population confirmed that a very prolonged BP treatment (i.e., years of monthly pamidronate or zoledronic acid) is a major risk factor for ONJ. The two ONJ cases observed in the “*screening*” group after zoledronic acid (one after three infusions in a patient with implant-related infection, and one after 18 administrations without apparent dental risk factors) are representative of the possible ONJ risk in patients receiving a potent BP without pre-start oral care: ONJ can clinically appear early if an untreated predisposing oral disease is present, or mostly after 12–24 zoledronic acid infusions otherwise, in line with previous literature [14]. Finally, no ONJ case has been observed among 78 MM patients in the “*preventive*” group at this moment.

The main limit of our retrospective–prospective study is the (relatively) restricted follow-up time of the “*preventive*” group, in comparison with the “*historic*” group: we know that ONJ can appear years after the discontinuation of BP treatment [36]; Jackson reported that event as rare, but occurring even 69 months after the start of BP therapy [35]; consequently, oral monitoring of MM patients treated with BP must be continuous and registered.

### 3.3. ONJ in MM Patients: Uncertain Definition

A word of cautiousness on the classical definition of ONJ [37]: all of the ONJ literature in MM patients (including relevant data from the Myeloma IX Medical Research Council (MRC)trial) [34,35] is based on a purely clinical ONJ definition, originally proposed by a Task Force of the American Association of Maxillofacial Surgeons (AAOMS) in 2006 and published in 2007 [38], later confirmed in 2009 [39], based on the presence of bone exposure lasting at least 8 weeks in patients treated with bisphosphonates and never treated with radiation in the head and neck region. That definition was linked to a staging system based on the presence of clinical signs and symptoms, and it was also substantially approved by a task force of the American Society of Bone and Mineral Research [40]. The term BRONJ (bisphosphonate-related ONJ) entered use largely in the medical literature, also when the first cases of ONJ after treatment including antiangiogenic agents and denosumab began to be reported [41,42]. In the same years, clinical practice and literature [43,44] showed increasing evidence of symptomatic cases of BP-related jawbone alterations without frank bone exposure, so questioning the ONJ definition. In 2009, the AAOMS Task Force [39] did not modify the definition, but added a “stage 0” to classify cases with signs and symptoms of the jaws without bone exposure; the contradictory nature of that position paper was underlined by a series of researchers calling for a new larger definition to include the non-exposed ONJ, and consequently a new staging system [45,46]. In 2014, an AAOMS special committee released a third position paper [47] changing the term from BRONJ (bisphosphonate-related ONJ) to MRONJ (medication-related ONJ) in order to include cases arising after treatment with denosumab or antiangiogenic drugs and targeted therapies, without BPs; in that document, the definition of disease was enlarged to include cases with “bone that can be probed through an intraoral or extraoral fistula in the maxillofacial region that has persisted for longer than 8 weeks”, but the existence of a “stage 0 category” for patients with signs and symptoms without bone exposure was confirmed. On the other hand, an international task force recently confirmed the 2007 American Society for Bone and Mineral Research (ASBMR) definition without the AAOMS 2009 and 2014 amendments [48,49]. These controversies on definition are clearly of paramount importance as a possible cause of the incorrect estimation of ONJ incidence on clinical studies, as well as patient selection and follow-up duration [50], and they might also induce under-diagnosing of ONJ cases in clinical practice. A new definition and work-up system [51,52] can be implemented by the emergent role of imaging studies [53,54], crossing from a purely clinical definition to a definition based on clinical and imaging (above all, CT scan) figures: practically, including ONJ cases without bone exposure and with imaging significant jawbone alterations [51,52].

### 3.4. Conclusive Remarks

In conclusion, our experience seems to confirm the literature data suggesting that awareness of ONJ and proactive management of oral health—together with less intensive and prolonged BP therapy (recommended by more recent guidelines)—can reduce patient risk of ONJ in MM patients. Careful monitoring of the oral health of MM patients submitted to BP therapy—including signs different from bone exposure—is warranted to avoid underestimation or late diagnosis of ONJ that might expose patients to rare but life-threatening or lethal complications [55]. Further studies of tailored BP treatment could be of value in terms of both efficacy and safety of this inalienable therapy to maintain and ameliorate the quality of life of MM patients.

## Figures and Tables

**Table 1:** Patient Characteristics.

		Historic Group	Screening Group	Prevention Group
Number of Patients		21	20	78
Median Age		68 years (range 43–81)	69 years (range 55–85)	65 years (range 35–84)
Sex		6 male, 15 female	9 male, 11 female	37 male, 41 female
First line bisphosphonate	Pamidronate	15	7	41
	Zoledronic Acid	4	12	36
	Other	2	1	1
	Median N° of i.v. administrations	21 (range 1–75)	10 (range 1–52)	12 (range 1–18)
Second line bisphosphonate	Pamidronate	2	7	10
	Zoledronic acid	11	3	2
	Other (clodronic acid)	1	0	0
	Median N° of i.v. administrations	14 (range 2–9)	2.5 (range 1–16)	11 (range 2–23)
Third line bisphosphonate	Pamidronate	3	5	1
	Zoledronic acid	2	0	0
	Median N° of i.v. administrations	6 (range 1–24)	3 (range 1–21)	3 (range 1–24)
Total Median of i.v. administrations		40 (range 1–102)	18.5 (range 1–52)	13 (range 1–37)
Antiangiogenic Therapy	None	8	8	44
	Thalidomide	13	12	28
	Lenalidomide	0	0	4
	Other	0	0	2
ONJ		3	2	0

**Table 2:** ONJ Patient Characteristics.

Patient N°	Group	Dentistry Infections	Recent Tooth Extractions	DentistryImplants	Dentistry Prosthesis	Antiangiogenic Therapy	N° of i.v. Bisphosphonate Administrations
1	Historic	No	No	No	No	No	68 (only Pamidronate)
2	Historic	No	Yes	No	Yes	Thalidomide	102 (79 Pamidronate, 23 Zoledronic Acid)
3	Historic	No	No	Yes	No	No	75 (only Pamidronate)
4	Screening	No	No	Yes	No	No	3 (only Zoledronic Acid)
5	Screening	No	No	No	No	No	18 (only Zoledronic Acid)

**Table 3:** Incidence of BP-related ONJ in MM patients.

Paper	Patients	BP Type	Preventive Measures	ONJ Frequency (%)
Durie et al. [5]	904	Pam/Zol	no	6.8
Bamias et al. [6]	111	Pam/Zol/both	no	9.9
Dimopulos etal. [7]	202	Pam/Zol/both	no	7.4
Badros et al. [8]	90	Pam/Zol/both	no	24.4
Zervas et al. [9]	254	Pam/Zol/both	no	11.0
Calvos-Villas et al [10]	64	Zol/both	no	10.9
Tosi et al. [11]	259	Zol	no	3.5
Garcia-Garay et al. [12]	143	Pam/Zol/both	no	9.8
Dimopoulos et al. [15]	128	Zol	No (38)/Yes(90)	26.3 vs. 6.7
Present study	119	Pam/ Zol/both	No(41)/Yes(78)	12.2 vs. 0

BP = bisphosphonate, MM = Multiple Myeloma, Pam = pamidronate, Zol = zoledronic acid, both = pamidronate and zoledronic acid in sequence.

## References

[1] Kyle R.A., Rajkumar S.V. (2009). Criteria for diagnosis, staging, risk stratification and response assessment of multiple myeloma. Leukemia.

[2] Rajkumar S.V., Gahrton G., Bergsagel P.L. (2011). Approach to the treatment of multiple myeloma: A clash of philosophies. Blood.

[3] Kyle R.A., Yee G.C., Somerfield M.R., Flynn P.J., Halabi S., Jagannath S., Orlowski R.Z., Roodman D.G., Twilde P., Anderson K. (2007). American Society of Clinical Oncology 2007 clinical practice guideline update on the role of bisphosphonates in multiple myeloma. J. Clin. Oncol..

[4] Ruggiero S.L. (2009). Bisphosphonate-related osteonecrosis of the jaw (BRONJ): Initial discovery and subsequent development. J. Oral Maxillofac. Surg..

[5] Durie B.G., Katz M., Crowley J. (2005). Osteonecrosis of the jaw and bisphosphonates. N. Engl. J. Med..

[6] Bamias A., Kastritis E., Bamia C., Moulopoulos L.A., Melakopoulos I., Bozas G., Koutsoukou V., Gika D., Anagnostopoulos A., Papadimitriou C. (2005). Osteonecrosis of the jaw in cancer after treatment with bisphosphonates: Incidence and risk factors. J. Clin. Oncol..

[7] Dimopoulos M.A., Kastritis E., Anagnostopoulos A., Melakopoulos I., Gika D., Moulopoulos L.A., Bamia C., Terpos E., Tsionos K., Bamias A. (2006). Osteonecrosis of the jaw in patients with multiple myeloma treated with bisphosphonates: Evidence of increased risk after treatment with zoledronic acid. Haematologica.

[8] Badros A., Weikel D., Salama A., Goloubeva O., Schneider A., Rapoport A., Fenton R., Gahres N., Sausville E., Ord R. (2006). Osteonecrosis of the jaw in multiple myeloma patients: Clinical features and risk factors. J. Clin. Oncol..

[9] Zervas K., Verrou E., Teleioudis Z., Vahtsevanos K., Banti A., Mihou D., Krikelis D., Terpos E. (2006). Incidence, risk factors and management of osteonecrosis of the jaw in patients with multiple myeloma: A single-centre experience in 303 patients. Br. J. Haematol..

[10] Calvo-Villas J.M., Tapia Torres M., Govantes Rodríguez J., Carreter de Granda E., Sicilia Guillén F. (2006). Osteonecrosis of the jaw in patients with multiple myeloma during and after treatment with zoledronic acid. Med. Clin..

[11] Tosi P., Zamagni E., Cangini D., Tacchetti P., Di Raimondo F., Catalano L., D’Arco A., Ronconi S., Cellini C., Offidani M. (2006). Osteonecrosis of the jaws in newly diagnosed multiple myeloma patients treated with zoledronic acid and thalidomide-dexamethasone. Blood.

[12] Garcia-Garay C., Gonzalez-Garcia C., Juliana Majado M., Santos T., Borrego D. (2006). Osteonecrosis of the jaw in multiple myeloma patients. Experience of two hospitals. Blood.

[13] Kühl S., Walter C., Acham S., Pfeffer R., Lambrecht J.T. (2012). Bisphosphonate-related osteonecrosis of the jaws—A review. Oral Oncol..

[14] Campisi G., Fedele S., Fusco V., Pizzo G., Di Fede O., Bedogni A. (2014). Epidemiology, clinical manifestations, risk reduction and treatment strategies of jaw osteonecrosis in cancer patients exposed to antiresorptive agents. Future Oncol..

[15] Dimopoulos M.A., Kastritis E., Bamia C., Melakopoulos I., Gika D., Roussou M., Migkou M., Eleftherakis-Papaiakovou E., Christoulas D., Terpos E. (2009). Reduction of osteonecrosis of the jaw (ONJ) after implementation of preventive measures in patients with multiple myeloma treated with zoledronic acid. Ann. Oncol..

[16] Rosen L.S., Gordon D., Kaminski M., Howell A., Belch A., Mackey J., Apffelstaedt J., Hussein M., Coleman R.E., Reitsma D.J. (2001). Zoledronic acid versus pamidronate in the treatment of skeletal metastases in patients with breast cancer or osteolytic lesions of multiple myeloma: A phase III, double-blind, comparative trial. Cancer J..

[17] Rosen L.S., Gordon D., Kaminski M., Howell A., Belch A., Mackey J., Apffelstaedt J., Hussein M.A., Coleman R.E., Reitsma D.J. (2003). Long-term efficacy and safety of zoledronic acid compared with pamidronate disodium in the treatment of skeletal complications in patients with advanced multiple myeloma or breast carcinoma: A randomized, double-blind, multicenter, comparative trial. Cancer.

[18] Berenson J.R., Hillner B.E., Kyle R.A., Anderson K., Lipton A., Yee G.C., Biermann J.S., American Society of Clinical Oncology Bisphosphonates Expert Panel (2002). American Society of Clinical Oncology clinical practice guidelines: The role of bisphosphonates in multiple myeloma. J. Clin. Oncol..

[19] Hillner B.E., Ingle J.N., Chlebowski R.T., Gralow J., Yee G.C., Janjan N.A., Cauley J.A., Blumenstein B.A., Albain K.S., Lipton A. (2003). American Society of Clinical Oncology 2003 update on the role of bisphosphonates and bone health issues in women with breast cancer. J. Clin. Oncol..

[20] Marx R.E. (2003). Pamidronate (Aredia) and zoledronate (Zometa) induced avascular necrosis of the jaws: A growing epidemic. J. Oral Maxillofac. Surg..

[21] Ruggiero S.L., Mehrotra B., Rosenberg T.J., Engroff S. (2004). Osteonecrosis of the jaws associated with the use of bisphosphonates: A review of 63 cases. J. Oral Maxillofac. Surg..

[22] Durie B., Katz M., McCoy J., Crowley J. (2004). Osteonecrosis of the Jaw in Myeloma: Time dependent correlation with Aredia and Zometa use. Blood.

[23] Ruggiero S., Gralow J., Marx R.E., Hoff A.O., Schubert M.M., Huryn J.M., Toth B., Damato K., Valero V. (2006). Practical guidelines for the prevention, diagnosis and treatment of osteonecrosis of the jaw in patients with cancer. J. Clin. Oncol. Pract..

[24] Weitzman R., Sauter N., Eriksen E.F., Tarassoff P.G., Lacerna L.V., Dias R., Altmeyer A., Csermak-Renner K., McGrath L., Lantwicki L. (2007). Critical review: Updated recommendations for the prevention, diagnosis, and treatment of osteonecrosis of the jaw in cancer patients—May 2006. Crit. Rev. Oncol. Hematol..

[25] Advisory Task Force on Bisphosphonate-Related Ostenonecrosis of the Jaws, American Association of Oral and Maxillofacial Surgeons (2007). American Association of Oral and Maxillofacial Surgeons Position Paper on Bisphosphonate-Related Osteonecrosis of the Jaws. J. Oral Maxillofac. Surg..

[26] Campisi G., Di Fede O., Musciotto A., Lo Casto A., Lo Muzio L., Fulfaro F., Badalamenti G., Russo A., Gebbia N. (2007). Bisphosphonate-related osteonecrosis of the jaw (BRONJ): Run dental management designs and issues in diagnosis. Ann. Oncol..

[27] Mhaskar R., Redzepovic J., Wheatley K., Clark O.A., Miladinovic B., Glasmacher A., Kumar A., Djulbegovic B. (2010). Bisphosphonates in multiple myeloma. Cochrane Database Syst. Rev..

[28] Lacy M.Q., Dispenzieri A., Gertz M.A., Greipp P.R., Gollbach K.L., Hayman S.R., Kumar S., Lust J.A., Rajkumar S.V., Russell S.J. (2006). Mayo clinic consensus statement for the use of bisphosphonates in multiple myeloma. Mayo Clin. Proc..

[29] Durie B.G. (2007). Use of bisphosphonates in multiple myeloma: IMWG response to Mayo Clinic consensus statement. Mayo Clin. Proc..

[30] Imrie K., Stevens A., Makarski J., Email R., Meharchand J., Meyer R. (2007). The Role of Bisphosphonates in the Management of Skeletal Complications for Patients with Multiple Myeloma: A Clinical Practice Guideline.

[31] Terpos E., Sezer O., Croucher P.I., García-Sanz R., Boccadoro M., San Miguel J., Ashcroft J., Bladé J., Cavo M., Delforge M. (2009). The use of bisphosphonates in multiple myeloma: Recommendations of an expert panel on behalf of the European Myeloma Network. Ann. Oncol..

[32] Ripamonti C.I., Maniezzo M., Campa T., Fagnoni E., Brunelli C., Saibene G., Bareggi C., Ascani L., Cislaghi E. (2009). Decreased occurrence of osteonecrosis of the jaw after implementation of dental preventive measures in solid tumour patients with bone metastases treated with bisphosphonates. The experience of the National Cancer Institute of Milan. Ann. Oncol..

[33] Gimsing P., Carlson K., Turesson I., Fayers P., Waage A., Vangsted A., Mylin A., Gluud C., Juliusson G., Gregersen H. (2010). Effect of pamidronate 30 mg versus 90 mg on physical function in patients with newly diagnosed multiple myeloma (Nordic Myeloma Study Group): A double-blind, randomised controlled trial. Lancet Oncol..

[34] Morgan G.J., Davies F.E., Gregory W.M., Cocks K., Bell S.E., Szubert A.J., Navarro-Coy N., Drayson M.T., Owen R.G., Feyler S. (2010). First-line treatment with zoledronic acid as compared with clodronic acid in multiple myeloma (MRC Myeloma IX): A randomised controlled trial. Lancet.

[35] Jackson G.H., Morgan G.J., Davies F.E., Wu P., Gregory W.M., Bell S.E., Szubert A.J., Navarro Coy N., Drayson M.T., Owen R.G. (2014). Osteonecrosis of the jaw and renal safety in patients with newly diagnosed multiple myeloma: Medical Research Council Myeloma IX Study results. Br. J. Haematol..

[36] Del Conte A., Bernardeschi P., La Ferla F., Turrisi G., D’Alessandro M., Montagnani F., Fiorentini G. (2010). Bisphosphonate-induced osteonecrosis of the jaw 32 months after interruption of zoledronate in a patient with multiple myeloma. J. Oral Maxillofac. Surg..

[37] Fusco V., Bedogni A., Addeo A., Campisi G. (2016). Definition and estimation of osteonecrosis of jaw (ONJ), and optimal duration of antiresorptive treatment in bone metastatic cancer patients: Supplementary data from the denosumab extension study?. Support Care Cancer.

[38] American Association of Oral and Maxillofacial Surgeons (2007). Position paper on bisphosphonate-related osteonecrosis of the jaws. J. Oral Maxillofac. Surg..

[39] Ruggiero S.L., Dodson T.B., Assael L.A., Landesberg R., Marx R.E., Mehrotra B., American Association of Oral and Maxillofacial Surgeons (2009). Position paper on bisphosphonate-related osteonecrosis of the jaws—2009 update. J. Oral Maxillofac. Surg..

[40] Khosla S., Burr D., Cauley J., Dempster D.W., Ebeling P.R., Felsenberg D., Gagel R.F., Gilsanz V., Guise T., Koka S. (2007). Bisphosphonate-associated osteonecrosis of the jaw: Report of a task force of the American Society for Bone and Mineral Research. J. Bone Miner. Res..

[41] Estilo C.L., Fornier M., Farooki A., Carlson D., Bohle G., Huryn J.M. (2007). Osteonecrosis of the jaw related to bevacizumab. J. Clin. Oncol..

[42] Troeltzsch M., Woodlock T., Kriegelstein S. (2012). Physiology and Pharmacology of Nonbisphosphonate Drugs Implicated in Osteonecrosis of the Jaw. J. Can. Dent. Assoc..

[43] Junquera L., Gallego L. (2008). Non exposed bisphosphonate-related osteonecrosis of the jaws: Another clinical variant?. J. Oral Maxillofac. Surg..

[44] Mawardi H., Treister N., Richardson P. (2009). Sinus tracts—An early sign of bisphosphonate-associated osteonecrosis of the jaws?. J. Oral Maxillofac. Surg..

[45] Colella G., Campisi G., Fusco V. (2009). American Association of Oral and Maxillofacial Surgeons position paper: Bisphosphonate-Related Osteonecrosis of the Jaws-2009 update: The need to refine the BRONJ definition. J. Oral Maxillofac. Surg..

[46] Fusco V., Santini D., Armento G., Tonini G., Campisi G. (2016). Osteonecrosis of jaw beyond antiresorptive (bone-targeted) agents: New horizons in oncology. Expert Opin. Drug Saf..

[47] Ruggiero S.L., Dodson T.B., Fantasia J., Goodday R., Aghaloo T., Mehrotra B., O’Ryan F., American Association of Oral and Maxillofacial Surgeons (2014). American Association of Oral and Maxillofacial Surgeons position paper on medication-related osteonecrosis of the jaw—2014 update. J. Oral Maxillofac. Surg..

[48] Khan A.A., Morrison A., Hanley D.A. (2015). Diagnosis and management of osteonecrosis of the jaw: A systematic review and international consensus. J. Bone Miner. Res..

[49] Otto S., Marx R.E., Tröltzsch M., Ristow O., Ziebart T., Al-Nawas B., Groetz K.A., Ehrenfeld M., Mercadante V., Porter S. (2015). Diagnosis and Management of Osteonecrosis of the Jaw: A Systematic Review and International Consensus. J. Bone Miner. Res..

[50] Fusco V., Galassi C., Berruti A., Ciuffreda L., Ortega C., Ciccone G., Angeli A., Bertetto O. (2011). Osteonecrosis of the jaw after zoledronic acid and denosumab treatment. J. Clin. Oncol..

[51] Bedogni A., Fusco V., Agrillo A., Campisi G. (2012). Learning from experience. Proposal of a refined definition and staging system for bisphosphonate-related osteonecrosis of the jaw (BRONJ). Oral Dis..

[52] Schiodt M., Reibel J., Oturai P., Kofod T. (2014). Comparison of non-exposed and exposed bisphosphonate-induced osteonecrosis of the jaws: A retrospective analysis from the Copenhagen cohort and a proposal for an updated classification system. Oral Surg. Oral Med. Oral Pathol. Oral Radiol..

[53] Bedogni A., Fedele S., Bedogni G. (2014). Staging of jaw osteonecrosis requires computed tomography for accurate definition of the extent of bony disease. Br. J. Maxillofac. Surg..

[54] Fedele S., Bedogni G., Scoletta M. (2015). Up to a quarter of patients with osteonecrosis of the jaw associated with antiresorptive agents remain undiagnosed. Br. J. Oral Maxillofac. Surg..

[55] Mondello P., Pitini V., Arrigo C., Mondello S., Mian M., Altavilla G. (2014). Necrotizing fasciitis as a rare complication of osteonecrosis of the jaw in a patient with multiple myeloma treated with lenalidomide: Case report and review of the literature. Springerplus.

